# DRP1 haploinsufficiency attenuates cardiac ischemia/reperfusion injuries

**DOI:** 10.1371/journal.pone.0248554

**Published:** 2021-03-25

**Authors:** Laura Bouche, Rima Kamel, Sophie Tamareille, Gabriel Garcia, Camille Villedieu, Bruno Pillot, Naïg Gueguen, Ahmad Chehaitly, Juan Manuel Chao de la Barca, Justine Beaumont, Delphine Baetz, Michel Ovize, Hiromi Sesaki, Daniel Henrion, Pascal Reynier, Guy Lenaers, Fabrice Prunier, Delphine Mirebeau-Prunier

**Affiliations:** 1 Institut MITOVASC, CNRS UMR 6015 INSERM U1083, Université d’Angers, Angers, France; 2 Service de Cardiologie, CHU Angers, Angers, France; 3 Univ Lyon, CarMeN Laboratory, INSERM, Université Claude Bernard Lyon 1, Bron, France; 4 Département de Biochimie et Génétique, CHU Angers, Angers, France; 5 Service d’Explorations Fonctionnelles Cardiovasculaires & CIC de Lyons, Hôpital Louis Pradel, Hospices Civils de Lyon, Lyon, France; 6 Department of Cell Biology, Johns Hopkins University School of Medicine Hunterian 111, Baltimore, MD, United States of America; Virginia Commonwealth University, UNITED STATES

## Abstract

Mitochondrial dynamics is a possible modulator of myocardial ischemia/reperfusion injuries (IRI). We previously reported that mice partially deficient in the fusion protein OPA1 exhibited higher IRI. Therefore, we investigated whether deficiency in the fission protein DRP1 encoded by *Dnm1l gene* would affect IRI in *Dnm1l*^*+/-*^ mouse. After baseline characterization of the *Dnm1l*^*+/-*^ mice heart, using echocardiography, electron microscopy, and oxygraphy, 3-month-old *Dnm1l*^*+/-*^ and wild type (WT) mice were exposed to myocardial ischemia/reperfusion (I/R). The ischemic area-at-risk (AAR) and area of necrosis (AN) were delimited, and the infarct size was expressed by AN/AAR. Proteins involved in mitochondrial dynamics and autophagy were analyzed before and after I/R. Mitochondrial permeability transition pore (mPTP) opening sensitivity was assessed after I/R. Heart weight and left ventricular function were not significantly different in 3-, 6- and 12-month-old *Dnm1l*^*+/-*^ mice than in WT. The cardiac DRP1 protein expression levels were 60% lower, whereas mitochondrial area and lipid degradation were significantly higher in *Dnm1l*^*+/-*^ mice than in WT, though mitochondrial respiratory parameters and mPTP opening did not significantly differ. Following I/R, the infarct size was significantly smaller in *Dnm1l*^*+/-*^ mice than in WT (34.6±3.1% *vs*. 44.5±3.3%, respectively; p<0.05) and the autophagic markers, LC3 II and P62 were significantly increased compared to baseline condition in *Dnm1l*^*+/-*^ mice only. Altogether, data indicates that increasing fusion by means of *Dnm1l* deficiency was associated with protection against IRI, without alteration in cardiac or mitochondrial functions at basal conditions. This protection mechanism due to DRP1 haploinsufficiency increases the expression of autophagic markers.

## Introduction

Ischemic heart disease, and more specifically myocardial infarction (MI), is still the leading cause of morbidity and mortality in developed countries [1]. Timely reperfusion during myocardial infarction is crucial for the salvage of the ischemic myocardium, but paradoxically leads to ischemia-reperfusion injuries (IRI) that account for the final myocardial damage [2]. Due to their major cell functions, such as ATP synthesis, calcium homeostasis, and cell death/survival mechanisms, mitochondria are critical structures involved in IRI [3, 4]. Mitochondria are dynamic structures changing morphology by fission and fusion mechanisms under the action of proteins anchored in the inner or outer mitochondrial membranes [5]. This mitochondrial dynamics proves essential to maintaining the mitochondrial network integrity and appears of great relevance in IRI [6, 4]. Mitochondrial fusion orchestrated by optic atrophy 1 protein (OPA1) and mitofusin 1 and 2 (MFN1, 2) leads to elongated mitochondria, enabling exchanges of mitochondrial matrix proteins and mitochondrial DNA. In contrast, mitochondrial fission results in smaller and fragmented mitochondria and is orchestrated by the GTPase dynamin related protein-1 (DRP1). DRP1 is a cytosolic protein that translocates to the mitochondrial outer membrane following post-translational modifications. DRP1 oligomerization leads to mitochondrial fission through its interaction with outer mitochondrial membrane receptors, such as the human fission protein factor 1 (hFIS1), mitochondrial fission factor (Mff), and mitochondrial dynamics proteins of 49 and 51KDa (MiD49 and MiD51). Fission is an essential step in the mitophagy mechanism, enabling the segregation of unrecoverable mitochondria to initiate their elimination [7].

Myocardial IRI are associated with an imbalance in mitochondrial dynamics in favor of fission [8, 9]. Thus, modulating mitochondrial dynamics is a topic of intense research designed to limit IRI. In previous *in vivo* experiments, we observed a larger infarct size in mice with partial deficiency of the fusion protein Opa1 exposed to myocardial I/R than in that of wild-type [6]. This finding was consistent with an *in vitro* study in which simulated ischemia of the cardiomyoblast cell line H9C2 cells with Opa1 *shRNA* increased mitochondrial fragmentation and cell death [10]. Moreover, a mild Opa1 overexpression was found to protect the mice hearts from ischemic damage [11]. The opposite approach, consisting of inhibiting fission by pharmacologically targeting DRP1’s activity or *Dnm1l*’s expression [12, 13], provided controversial results. A cardioprotective effect has been observed in a few studies and attributed to the inhibition of mitochondrial permeability transition pore (mPTP) opening [9, 14]. Other studies reported increased IRI, which were attributed to mitochondrial autophagy inhibition [12].

To prevent cardiomyocytes death, damaged mitochondria should be removed or repair by adequate Mitochondrial fusion and fission. In this setting, it seemed interesting for us to study two genetics models of fusion (OPA1, precedent study) or fission (Dnml1, present study) haploinsuffisance. These two genetics models are not complete deletion of gene expression but only haploinsufisance and have no functional impact on the heart at three months. In precedent study with Opa1 haploinsufficiency, mice exhibited higher sensitivity to I/R with imbalance in dynamic mitochondrial Ca^2+^ uptake. Therefore, this study sought to investigate whether DRP1 deficiency influences myocardial IRI *in vivo* in a deficient *Dnm1l* mice model and mechanism in I/R injury which could be modulate by treatment.

## Methods

### Mouse model

DRP1 is encoded by the Dynamin 1 like gene (*Dnm1l*). Previously described heterozygous *Dnm1l* knockout (*Dnm1l* ^*+/-*^) mice (22) were obtained from Professor Hiromi Sesaki at the Johns Hopkins University. Mice were kept using standard light cycles, with food and water available ad libitum. All animal work was performed according to the European Community Guiding Principles in the care and use of animals (Directive 2010/63/UE; Décret n°2013–118). Authorizations to conduct animal experiments were obtained from the MENESR, *Ministère de l’Education Nationale de l’Enseignement Supérieur et de la Recherche* (APAFIS#4514–2016031415098201 v2).

### Echocardiography

Transthoracic echocardiography (TTE) was performed using an ultrasound system (Vivid 7 Pro, General Electric Medical Systems) on anesthetized mice (ketamine 60-70mg/Kg, intraperitoneally), as previously described [15]. Left ventricular end diastolic diameter (LVEDD), left ventricular end systolic diameter (LVESD), and fractional shortening (FS) were determined using two-dimensional (2D) M-mode echocardiography.

### Mitochondrial morphology by transmission electron microscopy

Samples of left ventricular (LV) papillary muscle were prepared for EM, as previously described [16]. In this muscle, the cardiac fibers are unidirectional (longitudinal fibers) which makes the mitochondrial area analysis comparable. At baseline or after 24h of reperfusion mice were sacrificed, the heart fixed and the papillary muscle isolated. Briefly, the hearts were fixed using a retrograde perfusion with 2% glutaraldehyde in cacodylate buffer (100mM sodium cacodylate and 2mM MgCl_2_; pH 7.3). Ultra-thin longitudinal sections of 60nm were cut and examined using an electron microscope, with 20,000x-magnified images by means of the software Image J, designed to assess mitochondrial number and area. In total, approximately 3900 mitochondrial area were quantified in both groups (WT and DRP1+/- mice) and were divided into quartiles in an ascending order (from the smallest mitochondrial area (0.011 μm) to the largest (4.5μm)). The percentage of mitochondria belonging to the WT or DRP1+/- group was counted in each of the divided quartiles.

### Respirometric investigation of mitochondrial function

Preparation of permeabilized cardiac fibers, previously described [17], was conducted in order to study mitochondrial function *in situ*. The respiratory rates were recorded at 30°C in 2mL glass chambers using a high-resolution Oxygraph respirometer equipped with a Clark oxygen sensor (Oroboros, Innsbruck, Austria) and analyzed by means of DATLAB Analysis software (OROBOROS, Austria).

Respiration was initiated with complex I-dependent substrates (5mM malate/ 2.5mM pyruvate). Complex I-coupled state 3 respiration was measured by adding saturating ADP (1mM), followed by 10mM succinate, enabling the full TCA cycle operation to occur, with maximal coupled respiration sustained by both complexes I+II. Next, 2.5μM rotenone was injected in order to obtain the complex II-coupled state 3 respiration. Oligomycin (8μg/mL) was then added to determine the uncoupled state 4 respiration. Finally, FCCP (1 μM) was added to induce the maximal uncoupled respiration, which allow the control of fiber bundles permeabilization by comparing this uncoupled respiration rate to the maximal phosphorylating one.

The second experiment was initiated with malate and palmitoylCoA/carnitine as substrates (2.5mM malate/ 40μM PalmitoylCoA/ 1mM carnitine), following which 1mM ADP was added in order to investigate the beta-oxidation under phosphorylating condition. Next, antimycin (2μg/mL) was added to inhibit complex III. The experiment was continued to assess complex IV-coupled state 3 respiration, following which complex IV inhibitors (1 mM KCN/ 2 mM azide) were added. Representative oxygraphic traces of the different experiments are shown in S1 Fig.

The last experiment was conducted in order to evaluate the coupling of oxidative phosphorylation with mitochondrial creatine kinase. Fibers were exposed to increasing ADP concentrations, either in the presence or absence of creatine (20mM), in order to stimulate the creatine kinase system; the ADP-stimulated respiration was plotted above basal oxygen consumption (V_0_) in order to determine the maximal respiration rate (V_max_).

### In vivo ischemia-reperfusion experiments and infarct size assessment

Overall, 3-month-old male *Dnm1l*^*+/-*^ mice and their littermates were submitted to myocardial I/R *in vivo*. *Dpr1*^*+/-*^ and WT mice were anesthetized by means of intraperitoneal sodium pentobarbital injections (80mg/Kg; Ceva Santé Animale) and received an injection of heparin (100IU/Kg, Heparine Choay^®^, Sanofi aventis, Paris, France) prior to thoracotomy. Myocardial I/R was achieved by temporarily occluding the left coronary artery, then releasing the occlusion as previously described (11,12). The duration of ischemia was 30 minutes, and that of reperfusion 24 hours.

To delimit the ischemic area-at-risk (AAR) and area of necrosis (AN), Evans Blue 4% (Sigma-Aldrich Co^®^, *Missouri*, *USA*) was injected through the left-ventricular apex. Thereafter, the heart sections were incubated with 2,3,5-triphenyltetrazolium chloride (TTC) (Sigma-Aldrich Co^®^, *Missouri*, *USA)* and fixed in 4% paraformaldehyde for 24 hours, as previously described [6]. The AN (white), AAR (not blue), and total left-ventricular (LV) areas from both sides of each section were measured using the software ImageJ 1.47. AAR/LV and AN/AAR were expressed as percentages, as previously reported [6].

### Western blot analysis

Three-month-old male and female *Dnm1l*^*+/-*^ mice and their littermates were employed. The methods applied for immunoblot preparation were previously described (11). Protein expression was assessed at basal condition or after the *in vivo* I/R procedure. Overall, 30–50μg of total proteins was separated by SDS-PAGE and transferred to a nitrocellulose or PVDF membrane. The membranes were incubated overnight at 4°C with primary antibodies, and thereafter, with appropriate secondary antibodies conjugated to horseradish peroxidase (Santa Cruz Biotechnology^®^, *Texas*, *USA*). The antibodies used consisted of OPA1, DRP1 (BD Transduction Laboratories^®^; 1/1000), MFN2 (Sigma-Aldrich^®^; 1/2000), FIS1 (Santa Cruz Biotechnology^®^; 1/500), LC3B (Enzo Life Sciences; 1/1000), p62 (Enzo Life Sciences; 1/1000), and GAPDH (Sigma-Aldrich^®^; 1/20000) was employed as a loading control.

### Metabolomic analysis

We applied a targeted, quantitative metabolomic approach to heart tissue extracts by using the Biocrates AbsoluteIDQ p180 Kit (Biocrates Life Sciences AG, *Innsbruck*, *Austria*), as previously described [18]. The ratio of short-chain acylcarnitines to free carnitine (C2+C3/C0) as a measure of overall beta-oxidation activity, and the ratio of long-chain acylcarnitines to free carnitine (C16+C18/C0) as activity of carnitine palmitoyltransferase 1 (CPT1) were quantified.

### Calcium Retention Capacity (CRC)

The mitochondrial permeability transition pore (mPTP) opening was assessed by mean CRC, as previously described [19]. In brief, extra-mitochondrial Ca^2+^ concentration was estimated using Calcium Green^TM^-5N (Life Technologies). Measurements were carried out on isolated cardiac mitochondria from *Dnm1l*^*+/-*^ after I/R were performed with or without the injection of cyclosporine A (10mg/kg) 10 minutes before the end of reperfusion. 250μg of mitochondrial proteins were added in 2mL of the incubation buffer (150mM sucrose, 50mM KCl, 2mM KH_2_PO_4_, 20mM tris/HCl, 5mM succinate and 0.25μM Calcium Green 5N). Mitochondria were gently stirred for 2 minutes at 25°C. Then, 10nmol CaCl_2_ pulses were applied every 90 seconds until mPTP opening. Finally, CRC was expressed as nmoles of Ca^2+^/mg of proteins (S2 Fig).

### Statistical analysis

Data have been expressed as mean±standard error of the mean (SEM). The statistical significance of the difference between the *Dnm1l* ^*+/-*^ and WT mice groups was estimated using the Student’s *t*-test or non-parametric Mann-Whitney test, as appropriate, with *p*-values <0.05 considered statistically significant.

## Results

### Anatomical characteristics and heart function of Dnm1l^+/-^ mice

**Fig 1:**
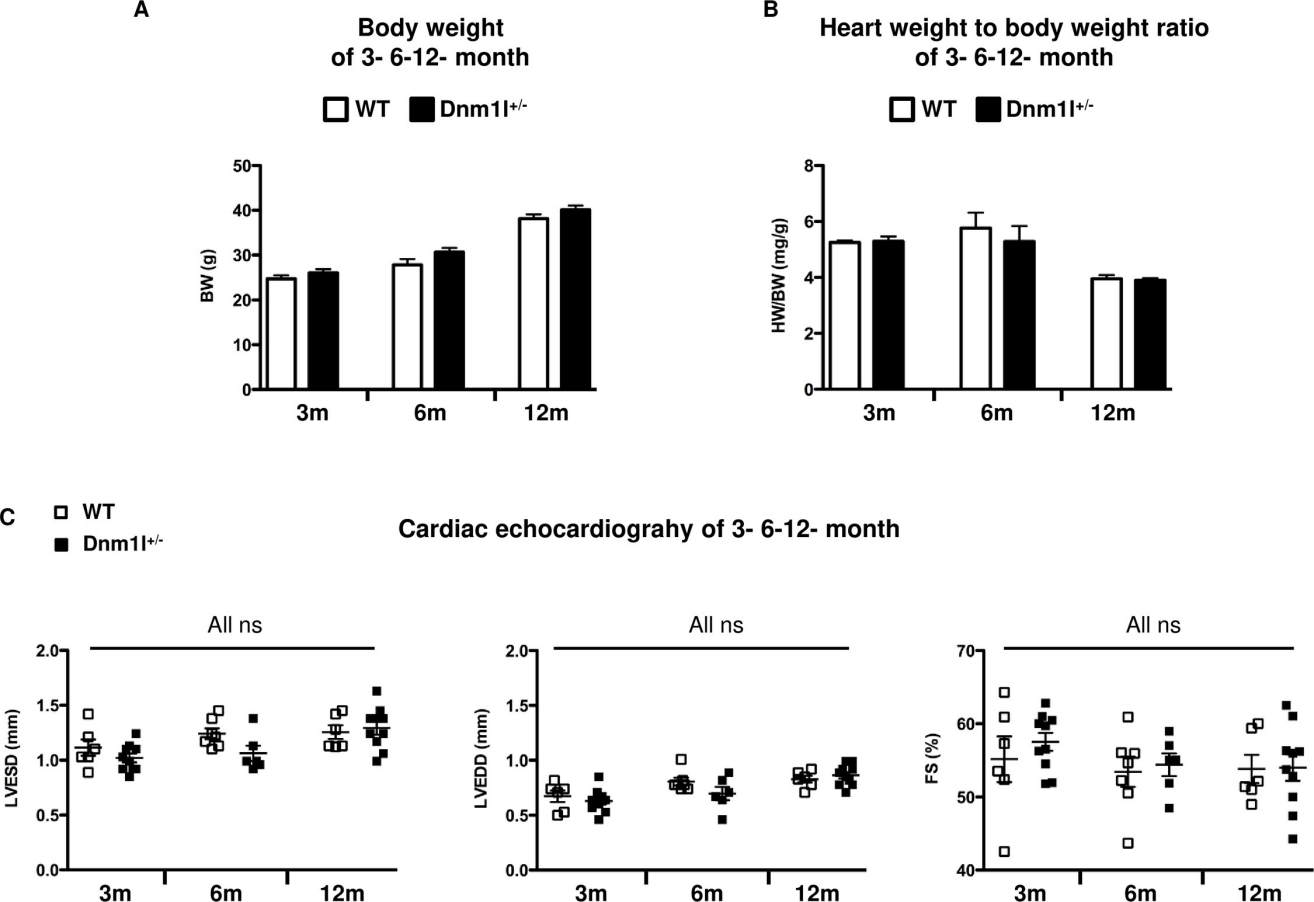
Anatomical characteristics and heart function of 3-, 6- and 12-month-old *Dnm1l+/-* and their wild type littermate (WT) mice. (A) Body weight (BW). (B) Heart weight to body weight (HW/BW) ratio. (C) Cardiac echocardiography, left: Left ventricle end-systolic volume (LVESD), middle: LV end-diastolic volume (LVEDD), right: LV fractional shortening (FS). Three months: n = 6–10 WT and n = 10 *Dnm1l*^*+/-*^; 6 months: n = 7–10 WT and n = 6–8 *Dnm1l*^*+/-*^; 12 months: n = 6–8 WT and n = 10 *Dnm1l*^*+/-*^.

We characterized the cardiac weight, LV size, and LV function in 3–6- and 12-month-old *Dnm1l*^*+/-*^ and WT mice. The heart rate recorded at the time of echocardiography was not significantly different between groups of the same age (591±30 bpm in WT *vs*. 574±15 bpm in *Dnm1l*^*+/-*^ at 3 months; 570±15 bpm in WT *vs*. 585±16 bpm in *Dnm1l* ^*+/-*^ at 6 months; 579±13 bpm in WT *vs*. 576±8 bpm in *Dnm1l* ^*+/-*^ at 12 months). Body, heart weights, and LV size were not significantly different between WT and *Dnm1l*^*+/-*^ mice of the same age (Fig 1A and 1B), nor were LVEDD and LVESD. Likewise, cardiac function represented by FS did not significantly differ at any time (55±2% in WT *vs*. 58±1% in *Dnm1l*^*+/-*^ at 3 months; 53±1% in WT *vs*. 54±1% in *Dnm1l*^*+/-*^ at 6 months; 54±2% in WT *vs*. 54±1% in *Dnm1l*^*+/-*^ at 12 months) (Fig 1C).

### In vivo I/R injury

**Fig 2:**
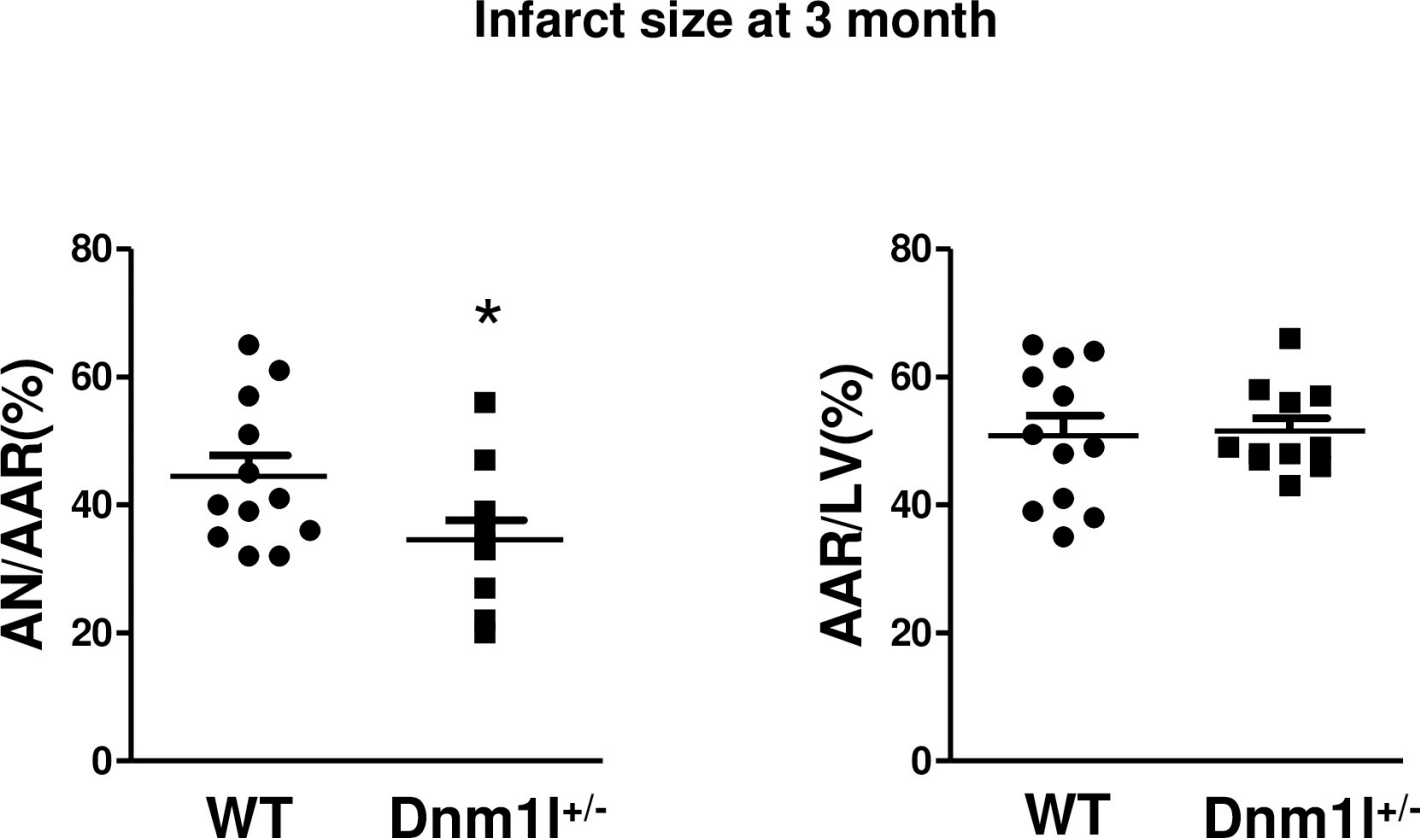
Histograms showing area necrosis (AN) as a percentage of area at risk (AAR) or AAR as a percentage of the total left ventricle (LV) area (n = 11-12/group) * *p*<0.05.

The 3-month-old *Dnm1l*^*+/-*^ and WT mice were subjected to 30-min myocardial ischemia followed by 24-hour reperfusion (Fig 2). The AN/AAR after I/R, evaluated using Evans Blue and TTC staining was significantly lower in *Dnm1l*^*+/-*^ mice than in WT (34.6±3.1% *vs*. 44.5±3.3%, respectively; *p*<0.05), whereas AAR/LV did not significantly differ between the two groups.

### Mitochondrial dynamics protein expression

**Fig 3:**
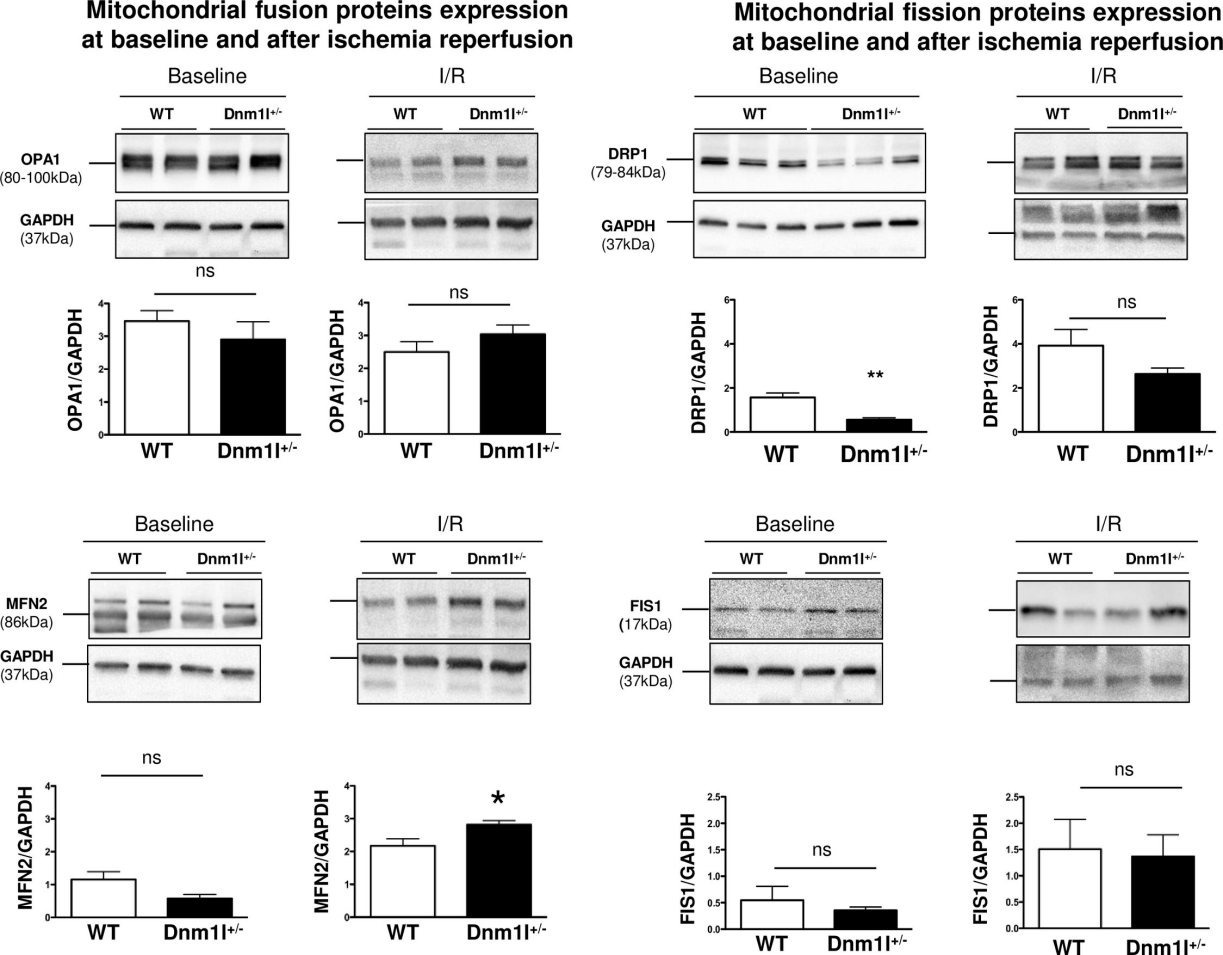
Baseline and post-ischemia/reperfusion (I/R) mitochondrial dynamics protein expression. OPA1, MFN2, DRP1, and FIS1 expression assessed by means of western blotting at baseline (n = 8-10/group) and after I/R (n = 4-10/group). Representative immunoblots and histograms showing quantifications are represented. GAPDH was used as a loading control. Values are expressed as mean ± SEM. * *p*<0.05.

Immunoblot analyses confirmed that cardiac DRP1 levels were 60% lower in 3-month-old *Dnm1l*^*+/-*^ mice than in WT (Fig 3). At baseline, cardiac levels of other proteins involved in mitochondrial dynamics, such as MFN 2, OPA1, and FIS1, were unaltered in *Dnm1l*^*+/-*^ mice compared to WT.

After a 30-min ischemia followed by a 24-hour reperfusion, DRP1 levels tended to be lower in *Dnm1l*^*+/-*^ mice, though the difference did not achieve statistical significance (3.7±0.5 in WT *vs*. 2.5±0.4 in *Dnm1l*^*+/-*^), whereas MFN2 levels were significantly increased in *Dnm1l*^*+/-*^ mice compared to WT (2.2±0.2 in WT *vs*. 2.8±0.1 in *Dnm1l*^*+/-*^). Furthermore, OPA1 and FIS1 were not significantly different in WT and *Dnm1l*^*+/-*^ mice (OPA1: 2.5±0.3 in WT *vs*. 3.0±0.3 in *Dnm1l*^*+/-*^; FIS1: 1.5±0.6 in WT *vs*. 1.4±0.4 in *Dnm1l*^*+/-*^) (Fig 3).

### Mitochondrial morphology

**Fig 4:**
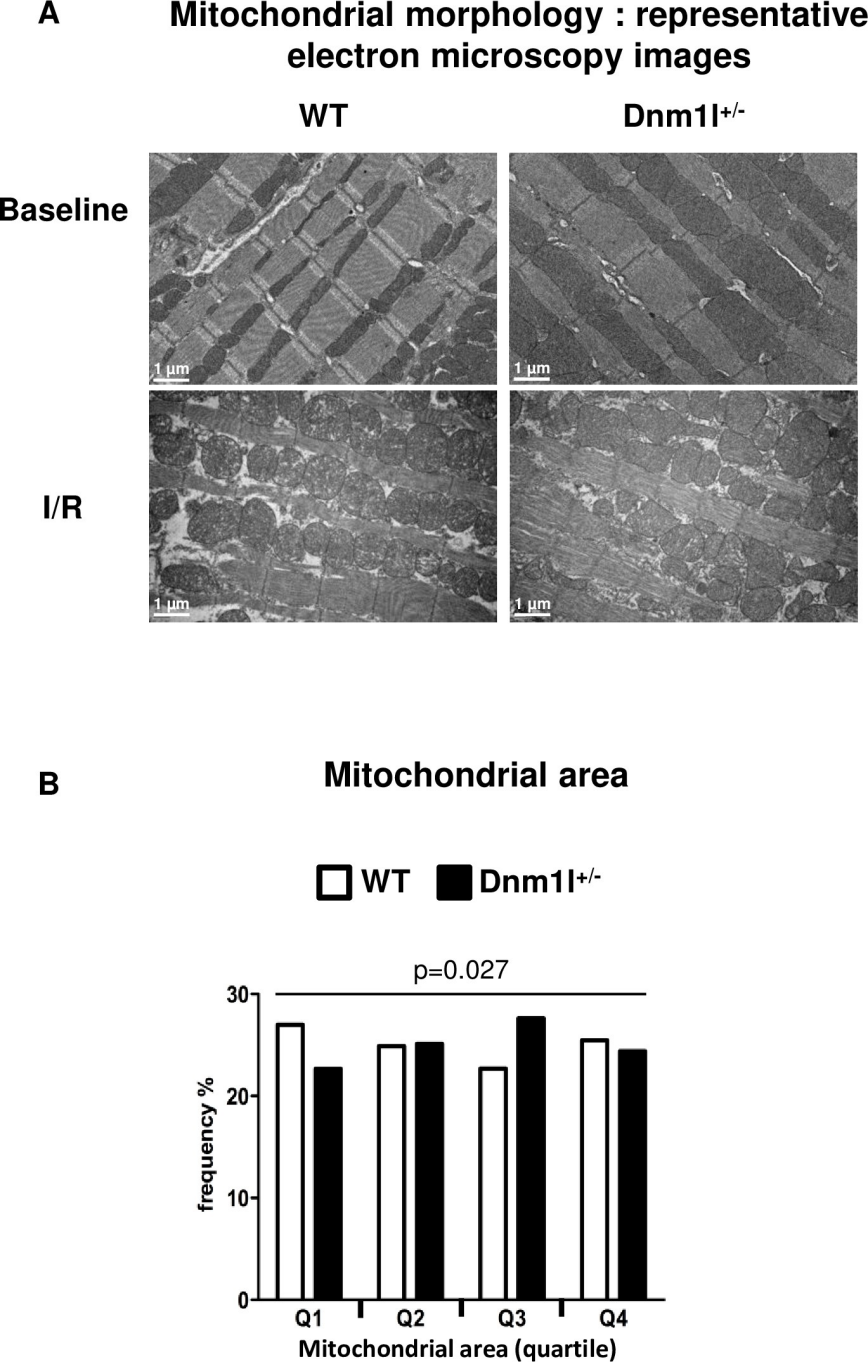
Mitochondrial morphology. (A) Representative electron microscopy (EM) images of left ventricle longitudinal sections at baseline and after 30 minutes of ischemia and 24 hours of reperfusion in 3-month-old *Dnm1l*^*+/-*^ and WT mice. Scale bar: 1 μm. (B) Mitochondrial frequency represented according to the quartile distribution of the mitochondrial area. Counting was performed on 60 representative EM fields per group (n = 4 per group).

Electron microscopy (EM) analyses of cardiac papillary muscle were performed at 3 months. At baseline, the number of mitochondria in hearts per field was not significantly different between *Dnm1l*^*+/-*^ and WT mice. However, the mitochondrial areas were more frequent in the first quartile in WT and in the third in *Dnm1l*^*+/-*^ mice, indicating that the mitochondria were enlarged in *Dnm1l*^*+/-*^ (Fig 4B).

We did not evaluate the mitochondrial size in papillary muscle from the ischemic area at 24h after reperfusion. In our model of I/R and in line with previous findings [20], ischemia induces mitochondrial swelling, cristae disruption which makes mitochondrial area assessment biased (Fig 4A).

### Mitophagy

**Fig 5:**
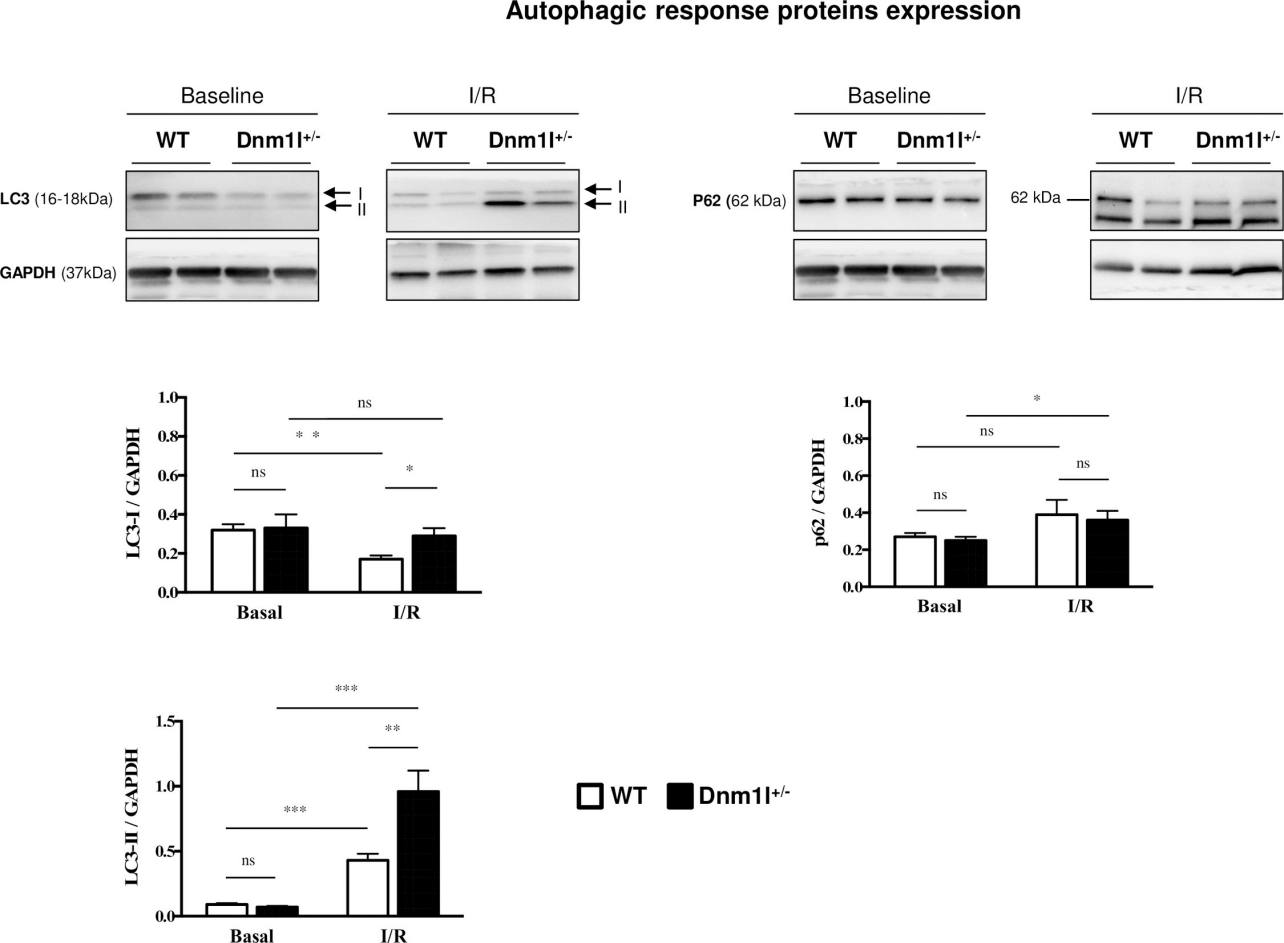
Representative immunoblot of autophagic response proteins. Autophagy levels were assessed by quantitative analysis of autophagy markers LC3-II and p62 in *Dnm1l*^*+/-*^ and WT mice at baseline and after ischemia/reperfusion (I/R). The results are expressed as ratios of protein band densities of LC3-II and p62, (n = 6–10 per group). GAPDH was used as a loading control. Values are mean ± SEM.

In the heart, we quantified the mitophagy protein levels, microtubule-associated proteins 1A/1B light chain 3B (LC3), and sequestosome-1 or ubiquitin-binding protein p62 from *Dnm1l*^*+/-*^ mice and WT, under basal conditions and after I/R. Under basal conditions, there was no significant difference in the protein level expression between *Dnm1l*^*+/-*^ and WT groups (Fig 5). After I/R, we observed that LC3 II protein level was increased compared to basal condition in both group WT (0.09±0.01 at baseline *vs*. 0.43±0.05 after I/R p<0.001) and *Dnm1l*^*+/-*^ mice (0.07±0.01 at baseline *vs*. 0.96±0.16 after IR p<0.001). This increase was significantly higher in *Dnm1l*^*+/-*^ mice (p<0,001) than in WT (p<0,01). P62 protein level was significantly increased after I/R in the *Dnm1l*^*+/-*^ mice only (0.25±0.02 at baseline *vs*. 0.36±0.05 after IR p = 0.038).

### Mitochondrial function

#### Cardiac energy metabolism of Dnm1l ^+/-^ mice

**Fig 6:**
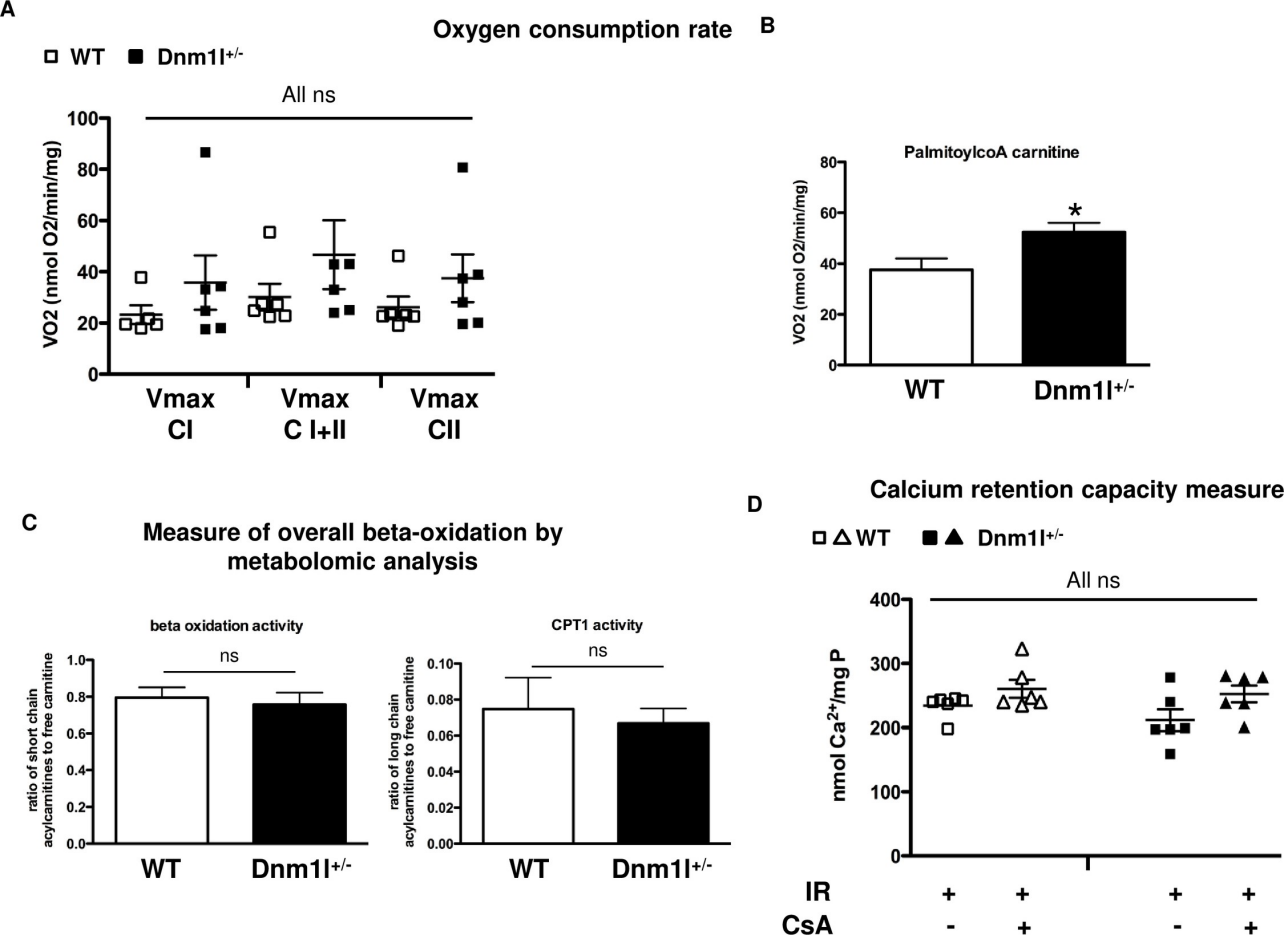
Cardiac energy metabolism and mPTP function. (A) Oxygen consumption rate of permeabilized left ventricular fibers in the presence of 1mM ADP (V_max_) with 5 mMmalate and 2.5mM pyruvate (complex I, CI), 10mM succinate (both CI and complex II CI + II), and after complex I inhibition with 2.5μM rotenone (CII). Values are expressed as mean ± SEM, (n = 5–6 per group). B) Oxygen consumption rate with 1mM ADP and 2.5mM malate and 40μM palmitoylCoA/1mM carnitine. Values are expressed as mean ± SEM, n = 7–8 per group; * p<0.05. (C) Metabolomic analysis of the cardiac tissue in 3-month-old *Dnm1l*^*+/-*^ and control WT mice. n = 10 per group. Left: ratio of short chain acylcarnitines to free carnitine (C2+C3/C0) represents a measure of overall beta-oxidation activity. Right: ratio of long chain acylcarnitines to free carnitine (C16+C18/C0) represents the activity of carnitine palmitoyltransferase 1 (CPT1). (D) CRC was measured in mitochondria isolated from the areas at risk after a 45-min ischemia followed by a 15-min reperfusion. CRC measurement represented the Ca^2+^ level required for mitochondrial permeability transition pore (mPTP) opening. Extra-mitochondrial Ca^2+^ was detected using Calcium Green^TM^-5N (Life Technologies). Results were expressed as mean ± SEM nmol Ca^2+^ mg protein -1. (n = 6 per group). Mitochondria were isolated from 3-month-old *Dnm1l*^*+/-*^ and WT mice.

Next, we investigated whether any change in oxidative capacity at baseline could explain the protection in *Dnm1l*^*+/-*^ mice after I/R stress (S1 Fig). Neither the basal (state 2) nor the maximal phosphorylating (state 3) respirations significantly differed between the two groups when using complex I, complex II, or complexes I+II (fully operating TCA) substrates (Fig 6A). However, when using palmitoylcoA/carnitine as substrates, the maximal phosphorylating respiration was higher in *Dnm1l*^*+/-*^ mice than WT (37.5±3.8nmol O2/min/mg in WT *vs*. 52.3±3.0nmolO2/min/mg in *Dnm1l*^*+/-*^ (Fig 6B)), supporting a higher fatty acid oxidation capacity in *Dnm1l*^*+/-*^. Therefore, we performed metabolomic analysis on *Dnm1l*^*+/-*^ and WT heart tissues. There was no significant difference between the two groups in either beta oxidation activity (0.80±0.04 in WT *vs*. 0.76±0.05 in *Dnm1l*^*+/-*^) or CPT1 activity (0.08±0.01 in WT *vs*. 0.07±0.01 in *Dnm1l*^*+/-*^) (Fig 6C).

The sensitivity of mitochondrial respiration to ADP was estimated with and without creatine to study the coupling of oxidative phosphorylation with mitochondrial creatine kinase. The ratio of the apparent affinity (Km) for ADP without (K_mADP_) and with creatine (K_mADP+creat_) was assessed with no significant difference found between the two groups (8.2 ±2.3 in WT *vs*. 3.1±0.6 in *Dnm1l*^*+/-*^; *p* = 0.14) (S3 Fig).

#### Cardiac mPTP function of Dnm1l^+/-^ mice

CRC is taken as an indicator of susceptibility to mPTP opening following calcium overload. We measured CRC on mitochondria isolated from the area at risk of *Dnm1l*^*+/-*^ and WT mice. Following IR, CRC was not altered in either group. CsA, a selective mPTP inhibitor, tended to increase the CRC value in WT and *Dnm1l*^+/-^ mitochondria after I/R, though not significantly (Fig 6D).

## Discussion

Mitochondrial dynamics and myocardial IRI are thought to be closely related. Mitochondrial fission is observed during myocardial ischemia [8, 18, 21], and mice partially deficient in the fusion protein OPA1 were shown to exhibit higher IRI [6], while mild Opa1 overexpression has previously been revealed to be protective (12, 20). Given this context, targeting the mitochondrial fission protein DRP1 appears to be an attractive therapeutic strategy to limit IRI. Herein, we have reported that heterozygous deficient *Dnm1l* mice exhibited a lower IRI degree when exposed to myocardial ischemia-reperfusion *in vivo*.

Because complete KO *Dnm1l* was shown to induce embryonic death by Day E11.5 [22], we investigated a heterozygous deficient *Dnm1l* model (*Dnm1l*^*+/-*^) [22]. In these mice, we observed a 60%-decreased DRP1 cardiac expression, without any change in the other main proteins involved in mitochondrial dynamics. Importantly, no alteration of either cardiac morphology or function was found at the ages of 3, 6, and 12 months. In a conditional cardiac KO *Dnm1l* mice model, the *Dnm1l* downregulation induced at the age of 15 weeks provoked a lethal dilated cardiomyopathy 8 weeks later through altered mitochondrial respiration [12, 13]. The mitochondrial respiratory chain capacity, evaluated with either complex I, II, or with full operating TCA substrates in permeabilized cardiac fibers, was not found to be altered in our *Dnm1l*^*+/-*^ model, although the mitochondria capacity to oxidize free fatty acids, as evaluated using palmytoylCoA/carnitine as substrates, was found increased. The mechanism responsible for this alteration is still unclear. Alteration in the transport of long chain fatty acids through mitochondrial membrane does not appear to be involved, because the CPT1 activity remained unchanged. Interestingly, opposite results were found in *Opa1*^*+/-*^ mice, where cardiac mitochondria were revealed to be less able to oxidize lipids than those of their WT littermates. Mitochondrial fission, induced by stress, is known to be associated with a predominant glycolytic metabolism shift aimed to preserve the energy level [23]. Hence, one could suggest that the protective effect of increasing mitochondrial fusion is partly related to its association with increased mitochondrial lipid oxidation occurring at the time of reperfusion. More research is necessary to further understand the DRP1’s role in lipid oxidation regulation.

Due to its crucial role in cardiomyocyte death in the I/R setting, mPTP opening sensitivity appears to be a credible target that could be modulated by mitochondrial dynamics [10]. To date, there are discordant data regarding this potential interaction [9, 12, 24]. In cardiomyocytes with *Dnm1l* silencing using shRNA and in *Dnm1l*-KO mice hearts 4 weeks after tamoxifen injection, the mPTP opening was found to be accelerated by *Dnm1l* down-regulation. Nevertheless, since all experiments were performed without I/R, their extrapolation to IRI was limited [12]. On the contrary, pre-treatment of HL-1 cells or adult rat cardiomyocytes with the DRP1 inhibitor *Mdivi-1* delayed mPTP opening after simulated I/R [19]. In our model of partial *Dnm1l* expression down-regulation, mPTP opening sensitivity was found to be unaltered, as evaluated by mean *Calcium Retention Capacity* values. Therefore, the relationship between cardioprotection through DRP1 deficiency and mPTP opening sensitivity was not assumed.

Previous studies have shown pharmacological DRP1 inhibition to be able to protect the heart from IRI [12, 19, 24, 25]. In this respect, *Mdivi-1*, an inhibitor of DRP1’s activity, was able to prevent IRI in cellular and animal models, when administered as a pretreatment [10, 14, 18, 23]. On the contrary, a recent study revealed that partial or total cardiac downregulation of *Dnm1l* exacerbated myocardial IRI [12]. The lack of Dnm1l impaired autophagy, and then induced abnormal mitochondria accumulation and cardiomyocyte deaths increase. Surprisingly, other authors reported that *Dnm1l* ablation in adult mice cardiomyocytes resulted in hyper-mitophagy with mitochondrial-associated p62 and LC3 protein level upregulation [13]. Indeed, mitochondrial autophagy proves to be an essential regulator that contributes to strict quality control by maintaining intracellular homeostasis in cardiomyocytes. Both fission and fusion exert an impact on mitochondrial autophagy, with fusion allowing for mitochondrial content exchange and functional complementation, and fission enabling mitochondria enriched with damaged constituents to be isolated, thereby facilitating their degradation. During this process, DRP1 facilitates the degradation of damaged mitochondria [21], while MFN2 plays a crucial role in the fusion of autophagosomes with lysosomes, a critical step in autophagic degradation [26]. Hence, *Mfn2* deficiency in the heart was found to result in impaired mitophagy, mitochondrial dysfunction, and cardiac dysfunction [26, 27]. In our work, we have observed no difference between *Dnm1l*^*+/-*^ and WT mice under basal condition regarding LC3 and p62, two markers of mitophagy, which suggests that DRP1 protein level is high enough to maintain mitochondrial autophagy. After I/R, DRP1 happloinsufficiency observed under basal condition disappeared and DRP1 protein level was similar in *Dnm1l*^*+/-*^ and WT littermates, suggesting that DRP1 protein level increased more after I/R in *Dnm1l*^*+/-*^ mice. Moreover, LC3 II, P62 and MFN2, three proteins involved in mitophagy increased more in *Dnm1l*^*+/-*^ mice than in their WT littermates. This suggests that the cardioprotection observed in *Dnm1l*^*+/-*^ mice is related to two consecutive mechanisms: a lower expression of DRP1 protein at the time of ischemia, followed by a higher DRP increase after reperfusion, inducing more mitophagy.

### Limits

We have used a constitutive heterozygous deficient *Dnm1l* mice model designed to assess *in vivo Dnm1l*’s role in the development of IRI. As a consequence of this chronic *Dnm1l* deficiency, several unsuspected compensation mechanisms may have come into play, such as increased mitochondrial autophagy and lipid oxidation. Moreover, the results pertaining to the absence of effect on mPTP opening sensitivity must be interpreted with caution, as CsA used as a positive control did not affect mPTP opening under our experimental condition. Finally, cardioprotection was only investigated at 24 hours after I/R. It would be of interest to assess whether *Dnm1l* deficiency would similarly affect post-infarct cardiac remodeling at a more advanced timepoint.

## Conclusion

Increasing fusion by means of *Dnm1l* deficiency is associated with protection against IRI, without alteration in cardiac or mitochondrial functions at basal conditions. This cardioprotection mechanism appears to be related to an increased mitophagy process.

## Supporting information

S1 Fig The following trace shows the Oxygen concentration slope (nmol/ml) in blue and the Oxygen slope uncorrected [(pmol/(s*ml)] in pink over time (min). MP:Malate/Pyruvate, ADP: Adenosine diphosphate, Glut: Glutamate, Succ: Succinate, Rot: Rotenone, Cyt C: Cytochrome C, Oligo: Oligomycin, FCCP: Carbonyl cyanide-4-phenylhydrazone.
(PPTX)

S2 Fig CRC was performed on isolated cardiac mitochondria from the ischemic zone from *Dnm1l*^*+/-*^ and control WT mice after I/R, with or without the injection of cyclosporine A (10mg/kg) 10 minutes before the end of reperfusion. Calcium-green Fluorescence was expressed in arbitrary unit, F(A.U.) and represents extra-mitochondrial Ca^2+^. Ca^2+^ was added every 90 sec by increments of 10 nmoles per injection.
(PPTX)

S3 Fig (PPTX)

S1 Raw images (PPTX)

S2 Raw images (PPTX)
