# Supplementary material for: DRP1 haploinsufficiency attenuates cardiac ischemia/reperfusion injuries
Source: PLoS One. 2021 Mar 25;16(3):e0248554. doi: 10.1371/journal.pone.0248554 (PMC7993837; doi:10.1371/journal.pone.0248554)
Supplement: S1 Fig — MP:Malate/Pyruvate, ADP: Adenosine diphosphate, Glut: Glutamate, Succ: Succinate, Rot: Rotenone, Cyt C: Cytochrome C, Oligo: Oligomycin, FCCP: Carbonyl cyanide-4-phenylhydrazone. (PPTX) [file pone.0248554.s001.pptx]

## Slide 1
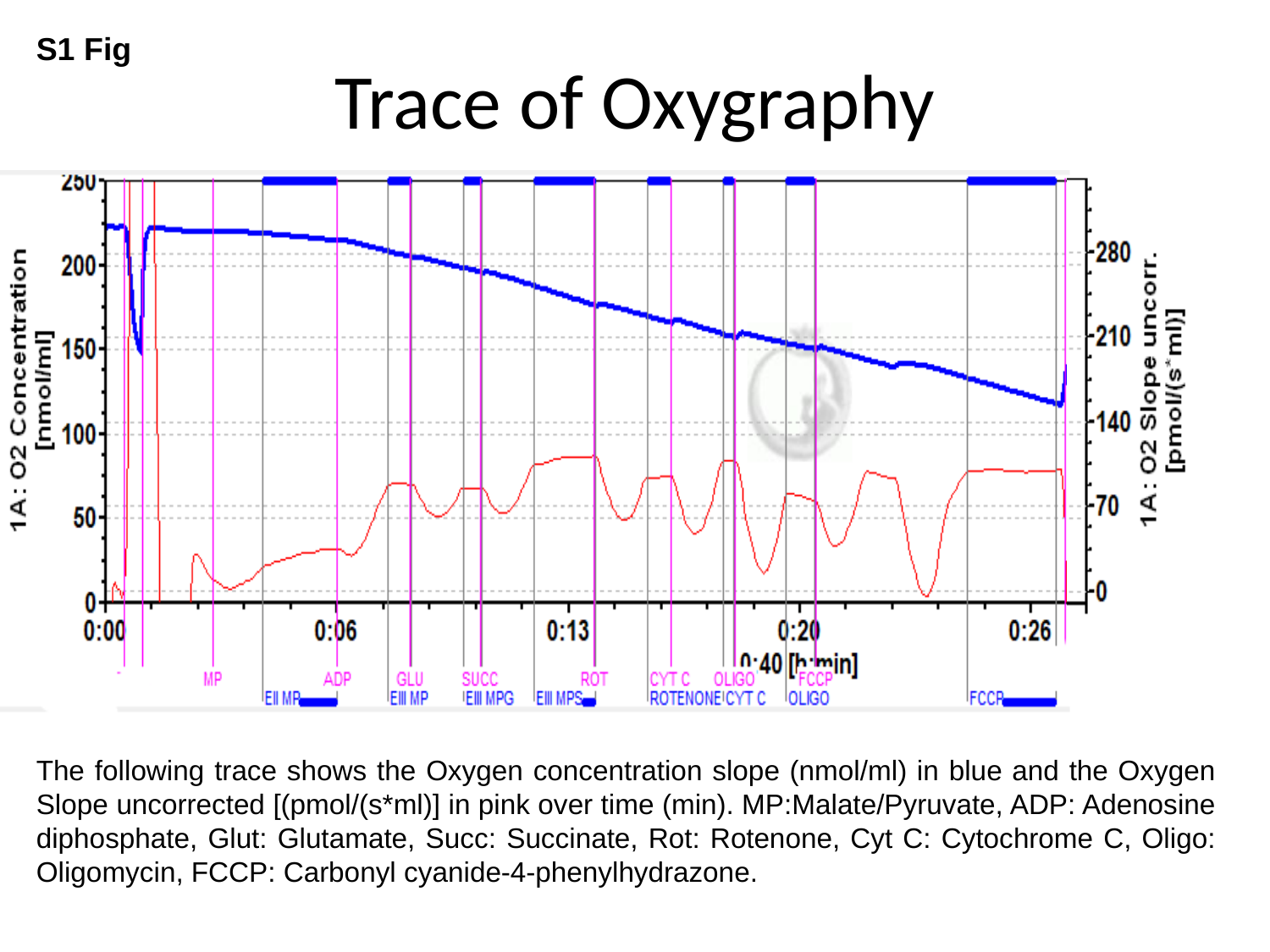

# Trace of Oxygraphy
S1 Fig
The following trace shows the Oxygen concentration slope (nmol/ml) in blue and the Oxygen Slope uncorrected [(pmol/(s*ml)] in pink over time (min). MP:Malate/Pyruvate, ADP: Adenosine diphosphate, Glut: Glutamate, Succ: Succinate, Rot: Rotenone, Cyt C: Cytochrome C, Oligo: Oligomycin, FCCP: Carbonyl cyanide-4-phenylhydrazone.
