# Supplementary material for: DRP1 haploinsufficiency attenuates cardiac ischemia/reperfusion injuries
Source: PLoS One. 2021 Mar 25;16(3):e0248554. doi: 10.1371/journal.pone.0248554 (PMC7993837; doi:10.1371/journal.pone.0248554)
Supplement: S2 Fig — Calcium-green Fluorescence was expressed in arbitrary unit, F(A.U.) and represents extra-mitochondrial Ca2+. Ca2+ was added every 90 sec by increments of 10 nmoles per injection. (PPTX) [file pone.0248554.s002.pptx]

## Slide 1
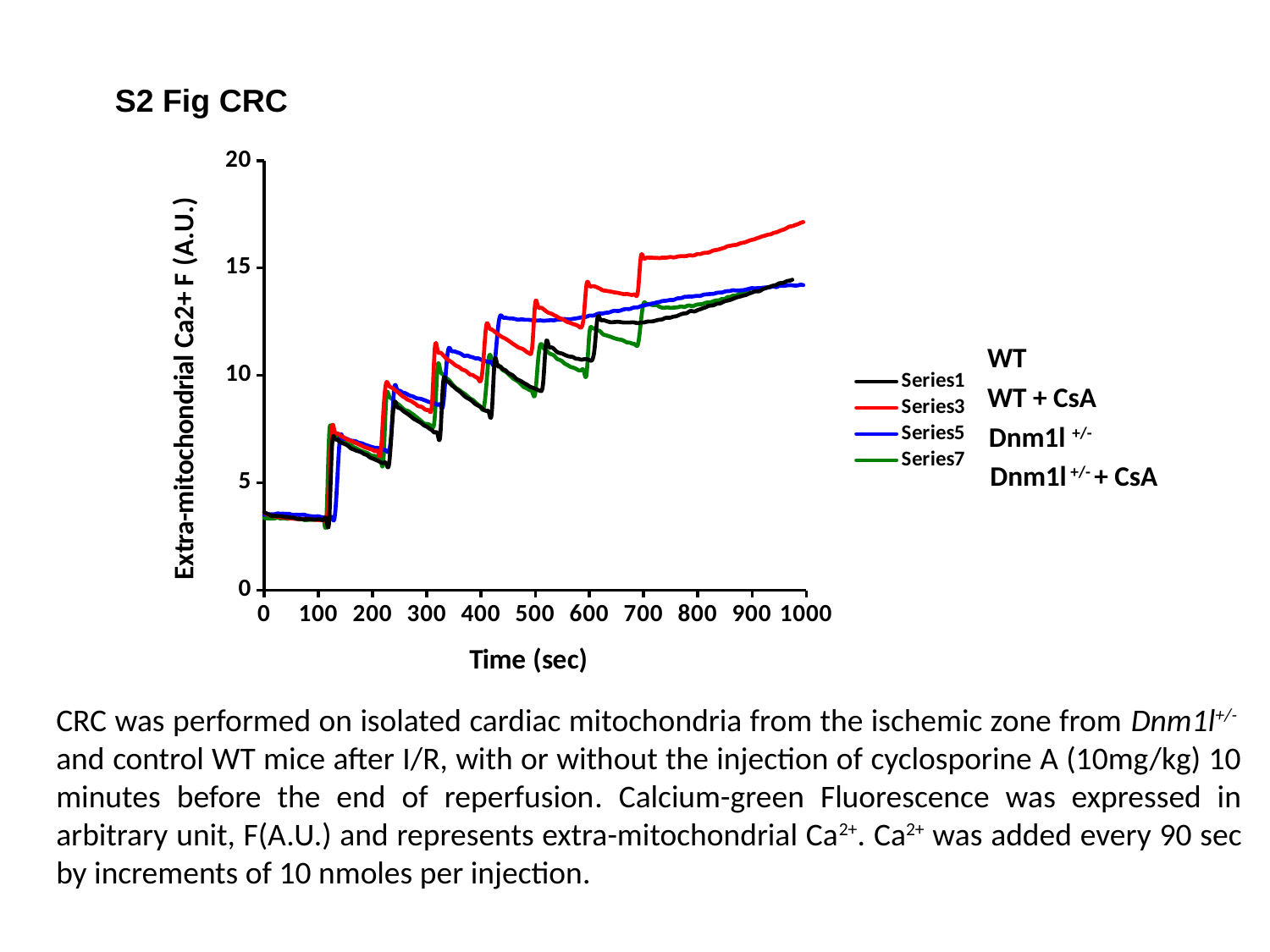

S2 Fig CRC
### Chart
| Category | | | | |
|---|---|---|---|---|WT
WT + CsA
Dnm1l +/-
Dnm1l +/- + CsA
CRC was performed on isolated cardiac mitochondria from the ischemic zone from Dnm1l+/- and control WT mice after I/R, with or without the injection of cyclosporine A (10mg/kg) 10 minutes before the end of reperfusion. Calcium-green Fluorescence was expressed in arbitrary unit, F(A.U.) and represents extra-mitochondrial Ca2+. Ca2+ was added every 90 sec by increments of 10 nmoles per injection.
