# Supplementary figures and images for: DRP1 haploinsufficiency attenuates cardiac ischemia/reperfusion injuries
Source: PLoS One. 2021 Mar 25;16(3):e0248554. doi: 10.1371/journal.pone.0248554 (PMC7993837; doi:10.1371/journal.pone.0248554)

## Slide 1
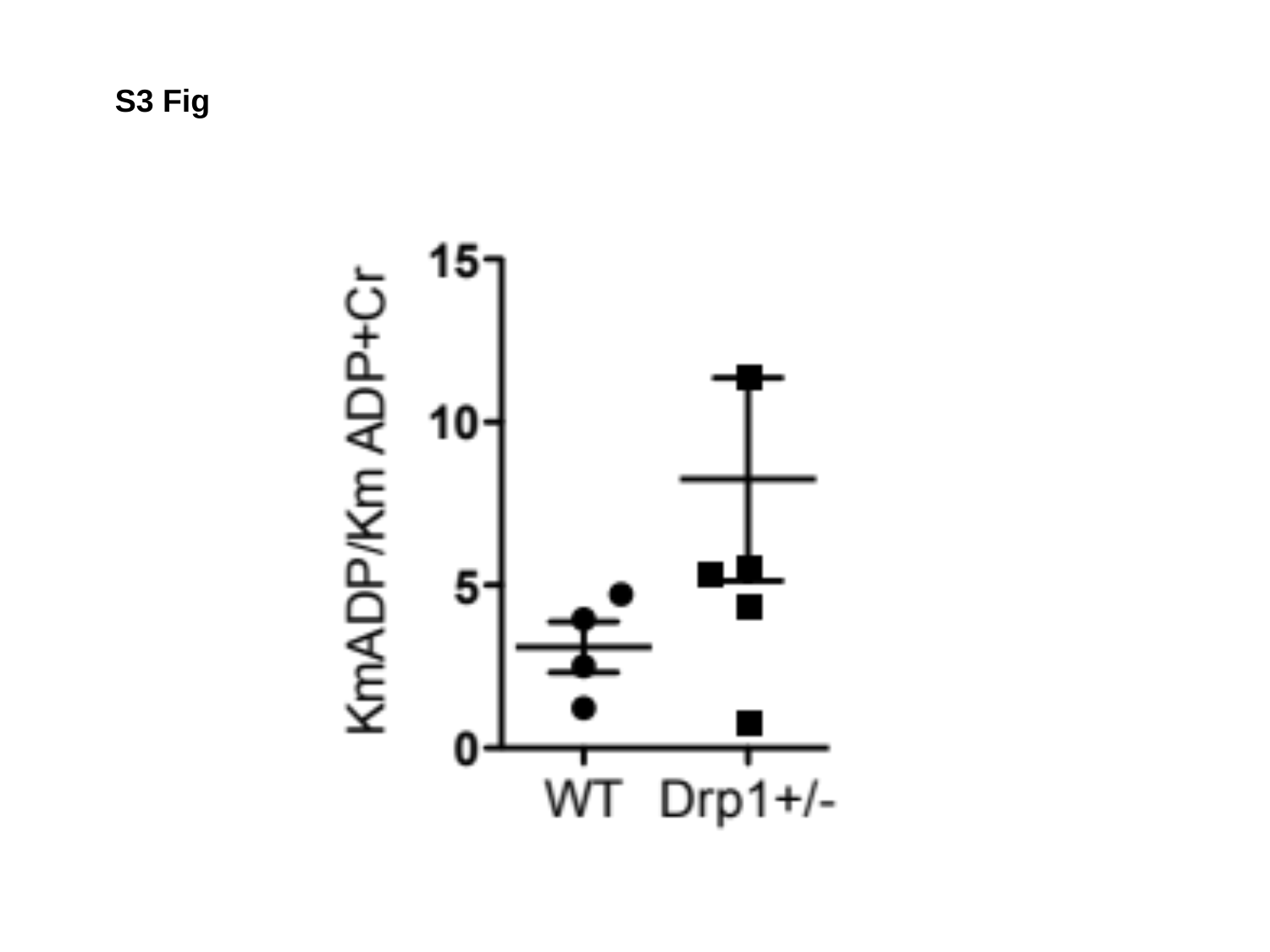

S3 Fig

Supplement: S3 Fig — (PPTX) [file pone.0248554.s003.pptx]
