# Supplementary material for: DRP1 haploinsufficiency attenuates cardiac ischemia/reperfusion injuries
Source: PLoS One. 2021 Mar 25;16(3):e0248554. doi: 10.1371/journal.pone.0248554 (PMC7993837; doi:10.1371/journal.pone.0248554)
Supplement: S1 Raw images — (PPTX) [file pone.0248554.s004.pptx]

## Slide 1
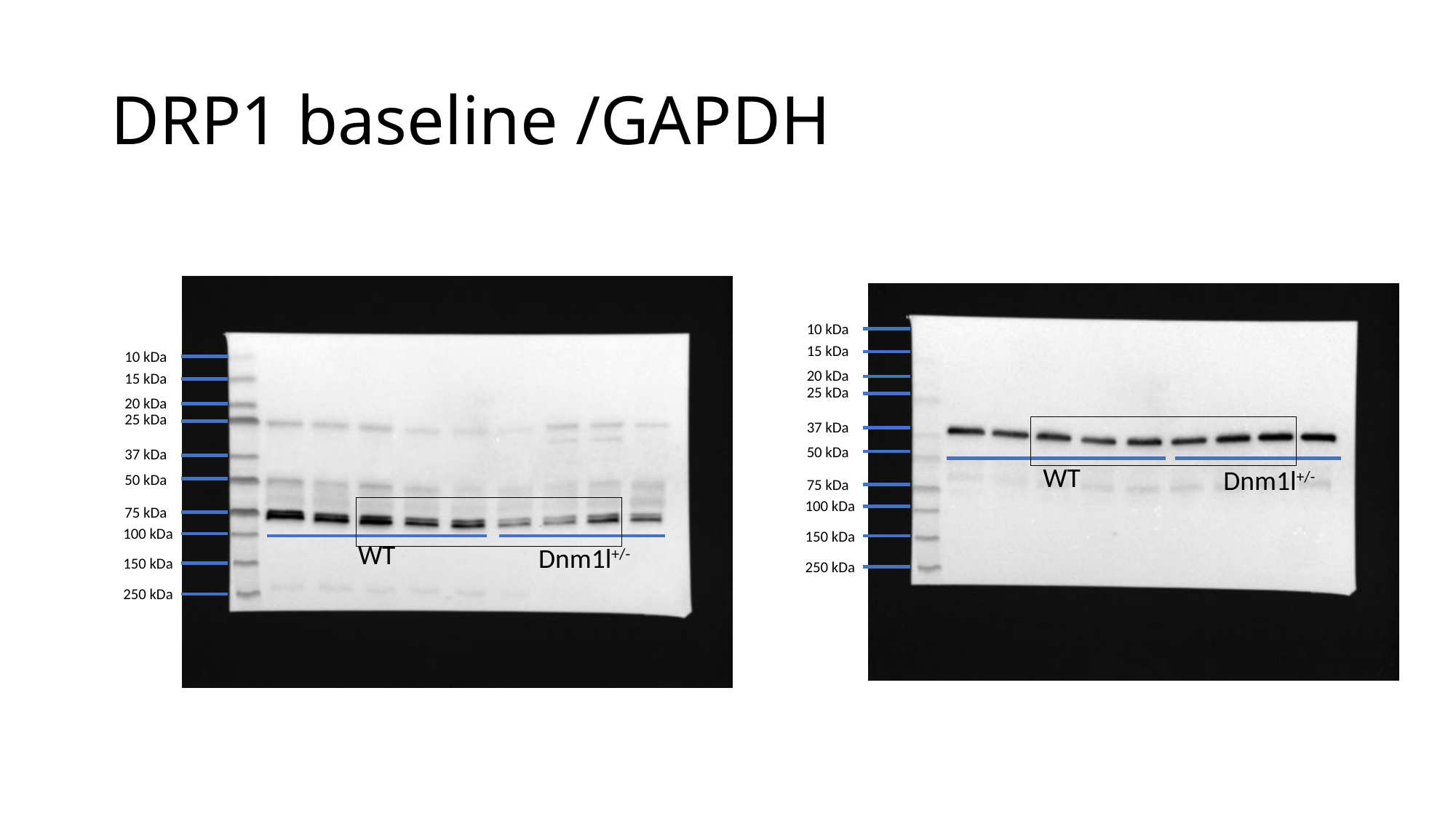

# DRP1 baseline /GAPDH
10 kDa
15 kDa
10 kDa
20 kDa
15 kDa
25 kDa
20 kDa
25 kDa
37 kDa
50 kDa
37 kDa
WT
Dnm1l+/-
50 kDa
75 kDa
100 kDa
75 kDa
100 kDa
150 kDa
WT
Dnm1l+/-
150 kDa
250 kDa
250 kDa

## Slide 2
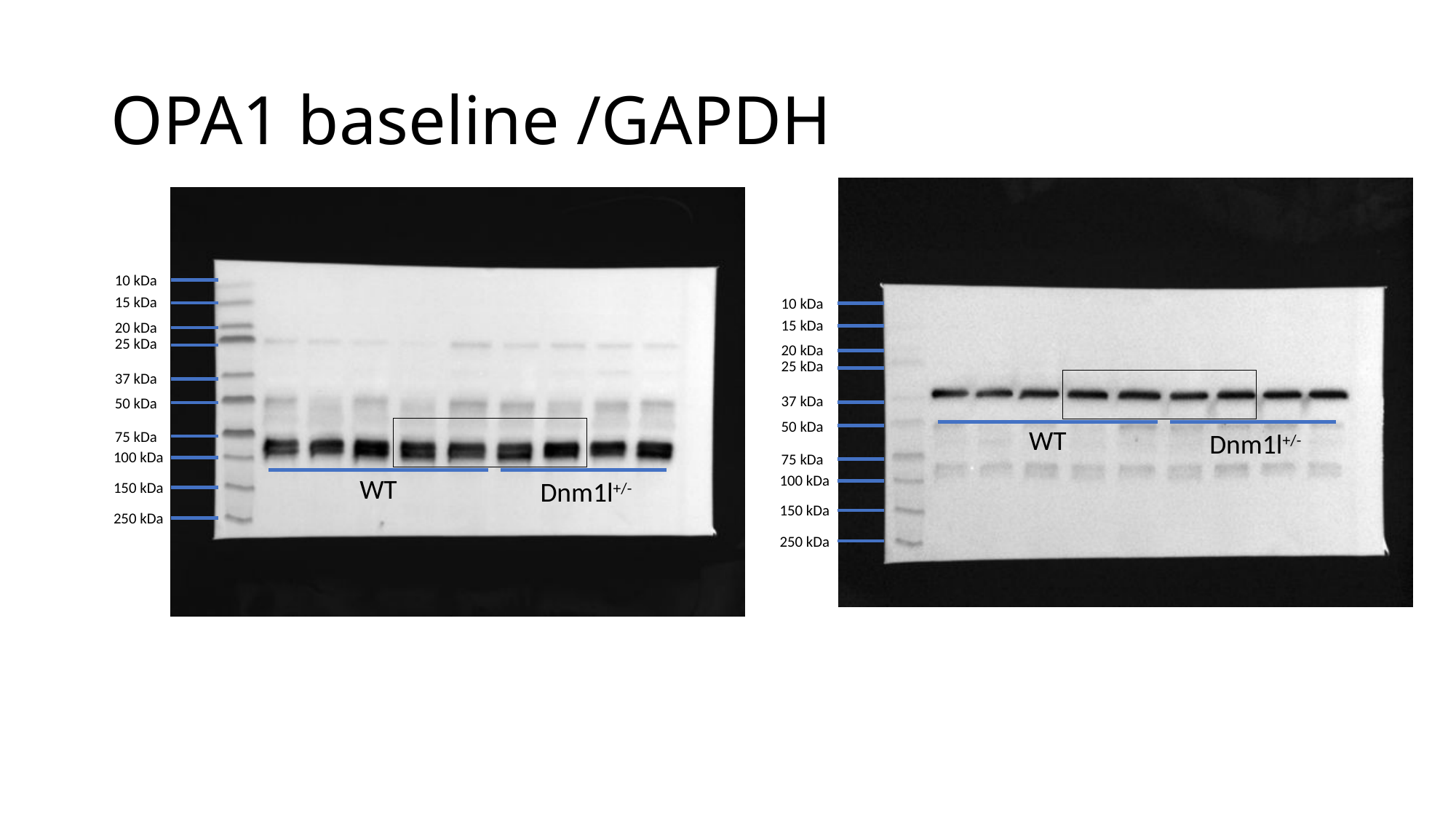

# OPA1 baseline /GAPDH
10 kDa
15 kDa
10 kDa
15 kDa
20 kDa
25 kDa
20 kDa
25 kDa
37 kDa
37 kDa
50 kDa
50 kDa
WT
Dnm1l+/-
75 kDa
100 kDa
75 kDa
100 kDa
WT
Dnm1l+/-
150 kDa
150 kDa
250 kDa
250 kDa

## Slide 3
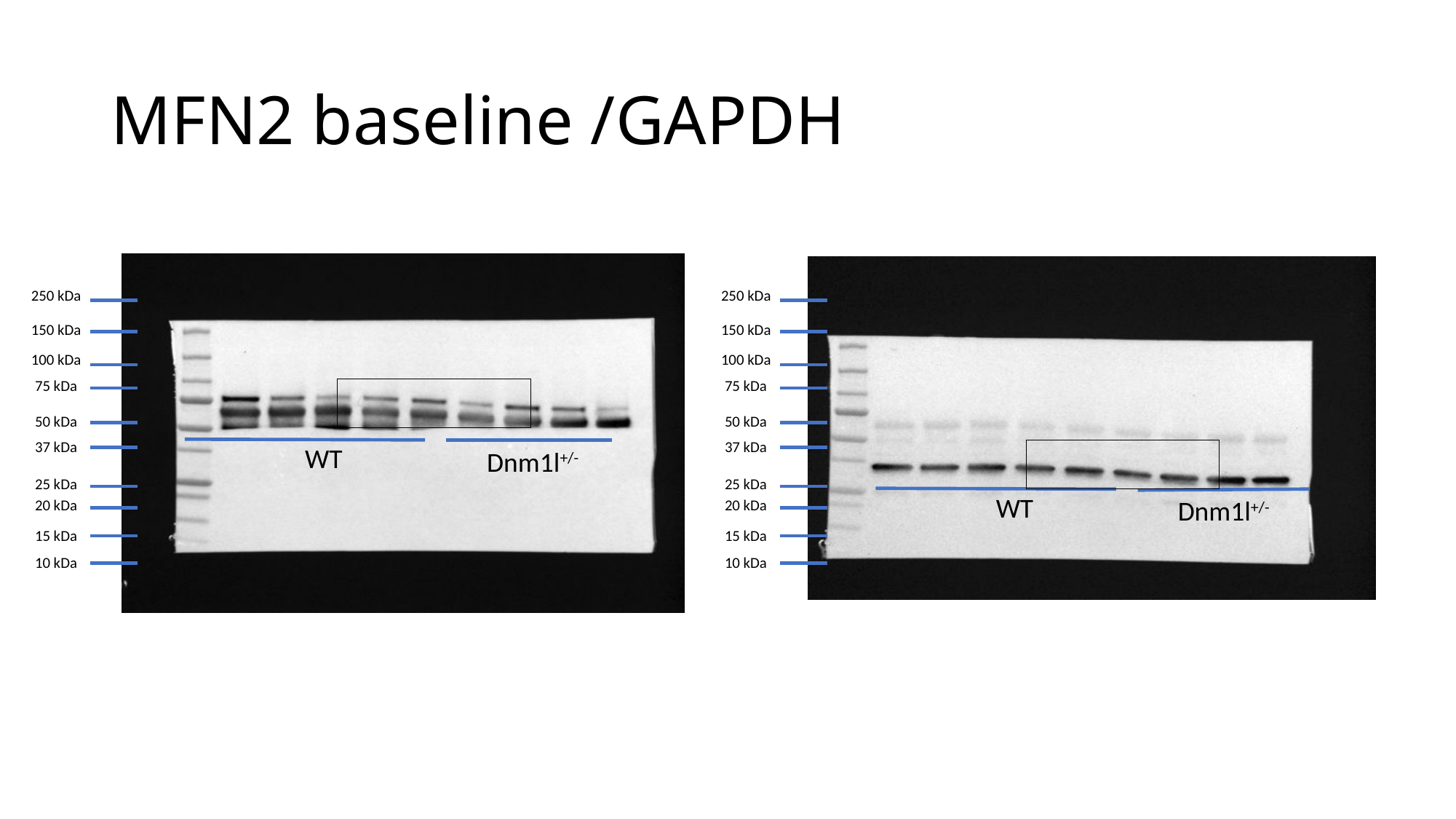

# MFN2 baseline /GAPDH
250 kDa
250 kDa
150 kDa
150 kDa
100 kDa
100 kDa
75 kDa
75 kDa
50 kDa
50 kDa
37 kDa
37 kDa
WT
Dnm1l+/-
25 kDa
25 kDa
WT
Dnm1l+/-
20 kDa
20 kDa
15 kDa
15 kDa
10 kDa
10 kDa

## Slide 4
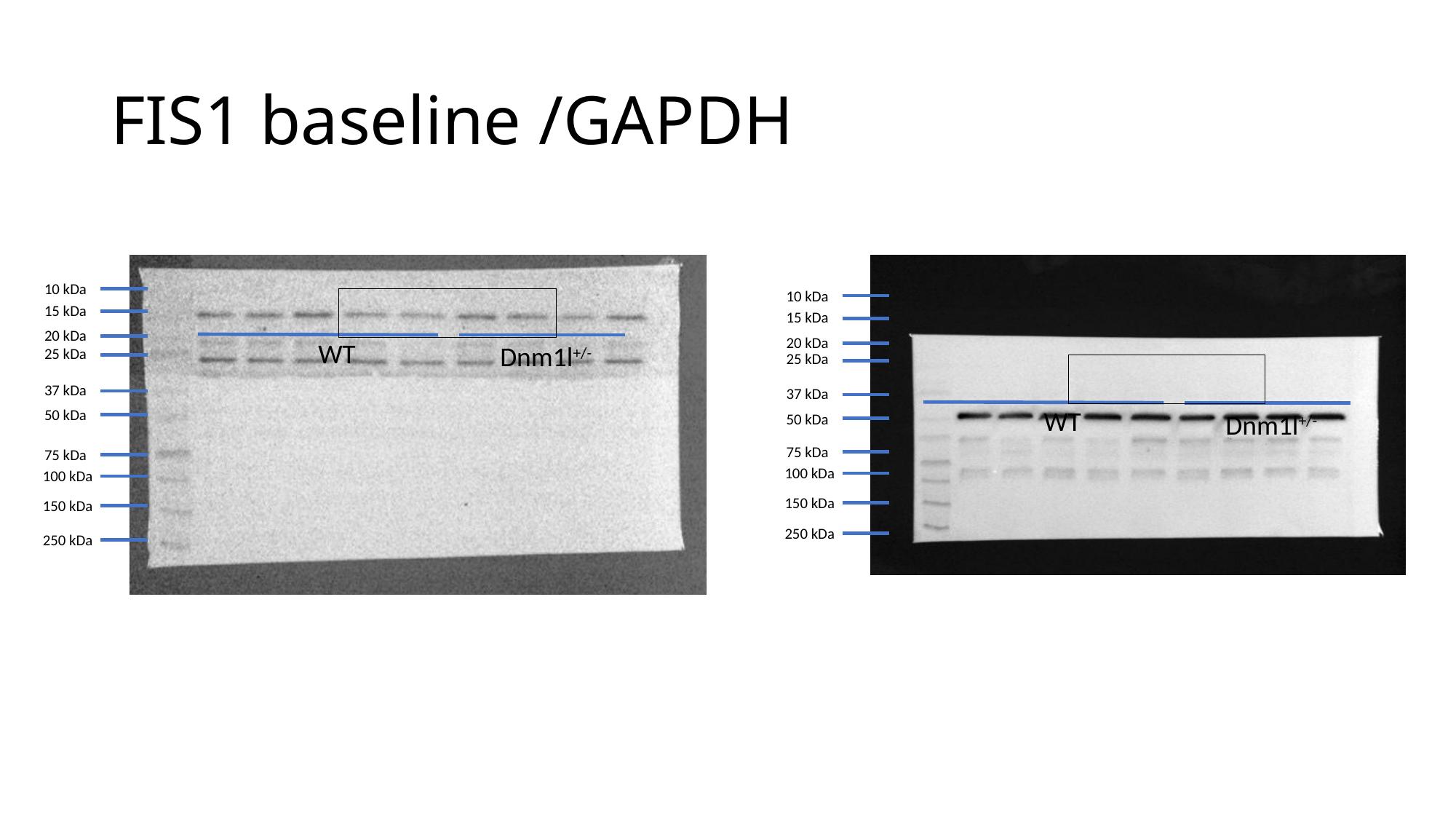

# FIS1 baseline /GAPDH
10 kDa
10 kDa
15 kDa
15 kDa
20 kDa
20 kDa
WT
Dnm1l+/-
25 kDa
25 kDa
37 kDa
37 kDa
WT
50 kDa
Dnm1l+/-
50 kDa
75 kDa
75 kDa
100 kDa
100 kDa
150 kDa
150 kDa
250 kDa
250 kDa

## Slide 5
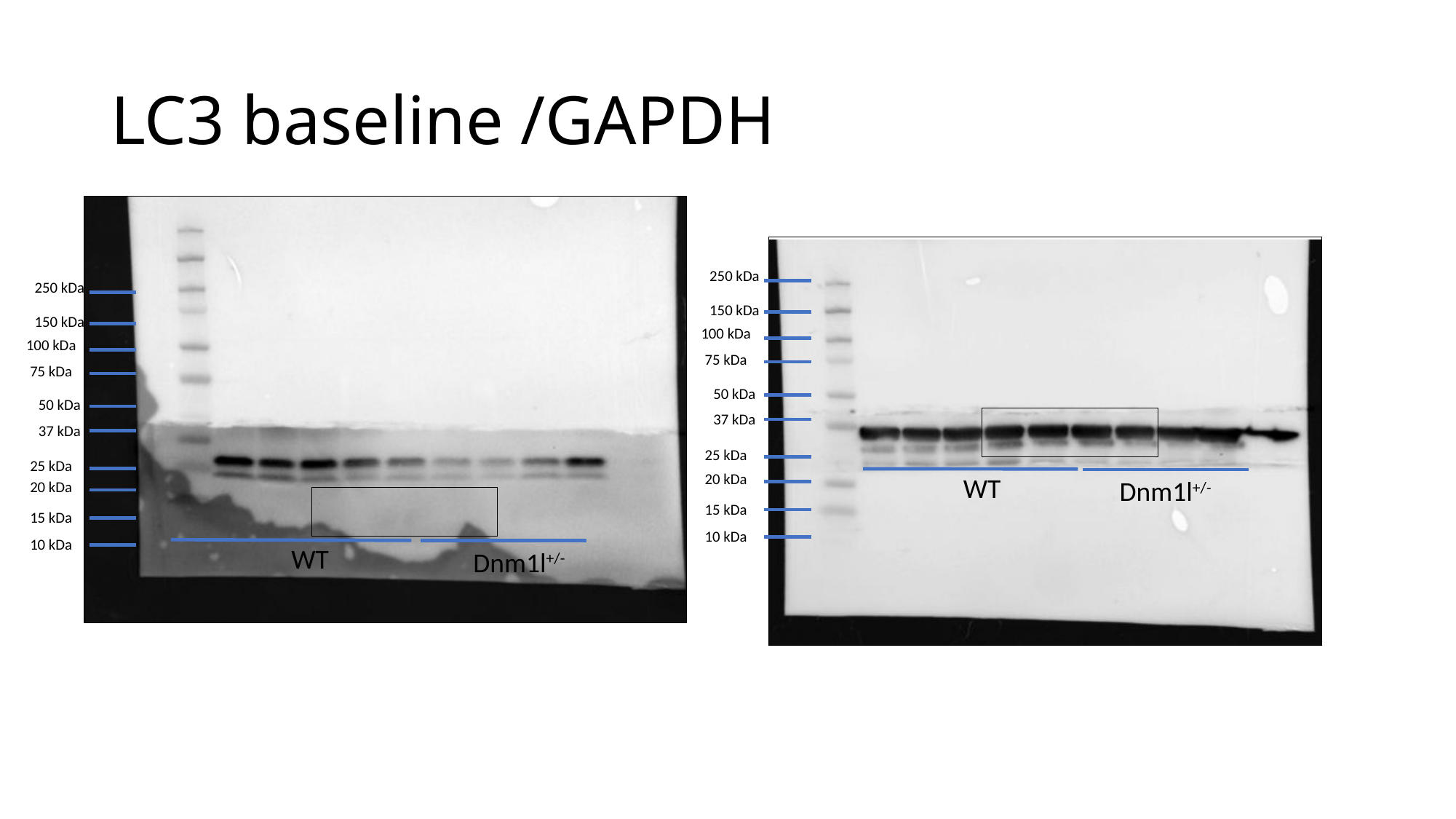

# LC3 baseline /GAPDH
250 kDa
250 kDa
150 kDa
150 kDa
100 kDa
100 kDa
75 kDa
75 kDa
50 kDa
50 kDa
37 kDa
37 kDa
25 kDa
25 kDa
20 kDa
WT
Dnm1l+/-
20 kDa
15 kDa
15 kDa
10 kDa
10 kDa
WT
Dnm1l+/-

## Slide 6
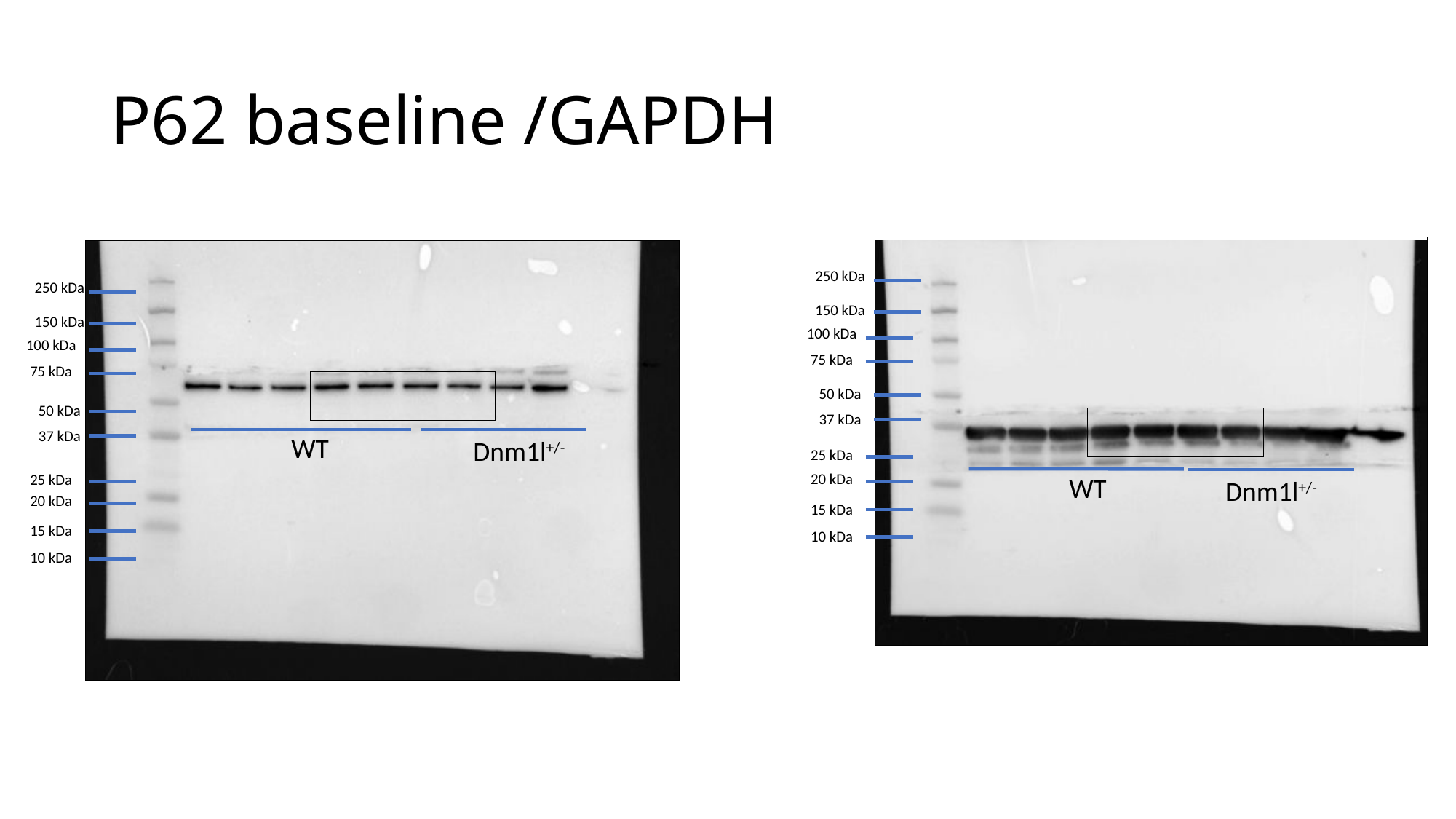

# P62 baseline /GAPDH
250 kDa
250 kDa
150 kDa
150 kDa
100 kDa
100 kDa
75 kDa
75 kDa
50 kDa
50 kDa
37 kDa
37 kDa
WT
Dnm1l+/-
25 kDa
20 kDa
25 kDa
WT
Dnm1l+/-
20 kDa
15 kDa
15 kDa
10 kDa
10 kDa
